# Reassessment of the Need for Asthmatic Patients for Biologic Treatment in a Tertiary Care Hospital

**DOI:** 10.7759/cureus.36288

**Published:** 2023-03-17

**Authors:** Sami M Alyami, Mosaad I Alshahwan, Hamad S Alshammari, Faisal M Abugamza, Sultan N Alotaibi, Omar A Abuoliat

**Affiliations:** 1 Medicine, King Abdul Aziz Medical City, Riyadh, SAU; 2 Pulmonary Medicine, King Saud Bin Abdul Aziz University for Health Sciences College of Medicine, Riyadh, SAU

**Keywords:** rhinosinusitis, gastroesophageal reflux, obstructive sleep apnea, biologics, asthma

## Abstract

Background: Asthma is a common chronic inflammatory airway condition. In difficult-to-treat asthma, poor control can be linked to multiple factors like the presence of uncontrolled comorbidities (e.g. gastroesophageal reflux and allergic rhinitis), as well as to poor inhaler use techniques and adherence. In this study we wanted to evaluate our severe asthma patients already on a biologic treatment with regard to presence of any of these factors.

Method: A questionnaire-based study, filled by investigators through direct interview with patients. We included all asthma patients on biologic treatment at King Abdul Aziz Medical City, Riyadh, KSA. Started in October 2020 and ended in December 2020. The questionnaire had a demographic section and sections for asthma symptoms, compliance, inhaler techniques, and comorbidities.

Result: Case series of N=38 severe asthma patients showed that majority had partially controlled or uncontrolled asthma (66%). Some 42% had intermediate/high risk for obstructive sleep apnea (OSA) based on the common screening tool “STOPBANG” score. Some 47% of our patients had uncontrolled gastro-esophageal reflux disease (GERD), and majority (80%) had uncontrolled allergic rhinitis. Only half of them demonstrated appropriate inhaler technique. And none of them was found exposed to asthma triggers at the time of interview.

Conclusion: Significant number of severe uncontrolled asthmatic patients were shown to be associated with at least one comorbid condition that might be interfering with patients’ improvement in asthmatic symptoms. By taking appropriate measures toward management and controlling of those comorbid conditions and also educating patients about technique to use inhalers might show notable improvement in asthmatic patients’ condition.

## Introduction

Asthma is a common chronic inflammatory condition that intermittently inflames and narrows the airways in the respiratory tract, and it affects more than 300 million individuals globally [1-2]. It develops as a consequence of the inflammatory reactions involving the neutrophils, eosinophils, macrophages, lymphocytes, and mast cells [3-4]. In difficult-to-treat asthma, poor control can be linked to poor adherence to inhaled therapy, incorrect inhaler technique, and coexisting conditions, including exposure to allergens and irritants [2]. Asthma is considered to be severe when control remains poor despite measures that adequately address medication delivery and comorbidities [5].

Adequate treatment of allergic asthma requires an array of different treatments to be administered for an indefinite amount of time; furthermore, before initiating a new drug therapy, practitioners should check the patient’s adherence with existing therapies, such as checking the inhaler techniques and eliminating triggering factors [6]. Causes behind insufficient asthma control are multifactorial which comprise medication access and compliance [7]. A diagnosis of severe asthma is established when all the comorbidities are identified and well specified.

Patients with severe asthma have persistent symptoms or frequent exacerbations that require repetitive glucocorticoid bursts, maintenance oral glucocorticoid therapy, or both, despite adequate treatment with high-dose inhaled glucocorticoids, long-acting β2-agonists, and long-acting muscarinic antagonists [8]. In these patients, add-on treatment, which may include biologic therapies (e.g., omalizumab and mepolizumab) is needed to reduce the disease burden.

There are many contributing factors to poorly controlled asthma. For example, poor inhaler techniques, which can compromise the medication efficacy, were found in 70%-80% of asthmatic patients [8-10]. In addition, 67% of physicians do not know how to illustrate the appropriate steps to use inhalers which can worsen the uncontrolled asthma [11]. Both active/passive electronic cigarette smoking can cause and exacerbate existing asthma [12]. Patient’s compliance can play a major role in guiding the treatment; for instance, youth and female patients were found better in adhering to the treatment plan than elderly and male patients [13]. While obesity is known to cause obstructive sleep apnea (OSA) and sleep-disordered breathing (SDB), obesity also plays a dose-related effect on asthma incidence among males and females, respectively [14-15].

Biologic treatment for severe asthma has got greater popularity among lung specialists, and data looking at justified prescribing habit are lacking. Therefore, the goal of this study was to evaluate our severe asthma patients who already are on a biologic treatment with regard to asthma medication compliance, asthma medication administration technique, existence of comorbidities known to interact with asthma, and how controlled are these comorbidities, as well as for environmental exposures.

## Materials and methods

The study was conducted at King Abdul Aziz Medical City in Riyadh Saudi Arabia (tertiary care hospital). A questionnaire was developed to collect information from the patients who agreed and consented to participate. Inclusion: All severe Asthma patients on biologics at King Abdul Aziz Medical City in Riyadh. Exclusion: any subjects less than 18 years of age, or using biologic treatment for diagnoses other than severe asthma.

Investigators interviewed the patients and filled the questionnaires themselves (started in October 2020 and ended in December 2020). The questionnaire had a demographic section and sections for asthma symptoms, compliance, inhaler techniques, and comorbidities. Then control of asthma symptoms was assessed based on the Global Initiative for Asthma in all of them at the time of interview. Those who had 20-25 points were considered having controlled asthma, those who scored 16-19 were considered partially controlled, and those who scored less than 16 points were considered in an uncontrolled asthma situation. Regarding OSA, patients were divided into three groups based on their STOPBANG score, where patient who scored 0-2 were considered having a mild risk to have OSA, those with scores 3-4 were considered at moderate risk, and those with 5-8 were considered having high risk to have an OSA. For GERD, patients were divided into two groups based on their GERD symptoms; if they have two or more days of symptoms they were given a score of 1, and if less they were given a score zero. Regarding compliance to asthma medications, they were asked 10 specific questions, and then were asked to demonstrate inhalers technique. Finally, they were asked to grade their confidence in using inhalers technique from 1 to 10. Data were entered in an encrypted excel sheet. Statistical Program for Social Sciences (SPSS) was used for data analysis. Frequencies and percentages were calculated for categorical variables, and cut off for significance was p value of less than 0.05.

## Results

An observational cross-sectional study of N=38 severe asthma patients (all were on biologic treatment), using Statistical Program for Social Sciences (SPSS) for analysis, showed that majority (63%) were female, and more than two third were overweight or obese, with remaining characters (educational level, age, and smoking status) listed in Table 1. Regarding asthma control, 34% had controlled asthma, 24% had partially controlled asthma, and 42% were with uncontrolled asthma. Regarding OSA risk assessed by STOPBANG scoring, 58% of the patients were with low OSA risk, and 24% with intermediate OSA risk, and 18% with high risk of having an OSA. Some 53% of our patients had controlled or no GERD, and majority (80%) of patients on biologics had uncontrolled allergic rhinitis/rhinosinusitis (Table 2). In 1-10 scoring, most of them (84%) scored themselves 8 and above, with regard to confidence level concerning how well they use their inhalers. However, when asked to demonstrate inhaler technique, only half of them (50%) demonstrated appropriate inhalers technique. Cross-tabulations between asthma control and comorbidities revealed no significant correlation, with the p-values above 0.05 (Tables 3-5). All of the patients were not found exposed to asthma triggers at the time of interview.

**Table 1:** General characteristics.

Characteristics		Number	Percent (%)
Gender	Male	14	37
	Female	24	63
BMI group	Normal weight	7	18
	Overweight	15	39
	obese	16	42
Educational level	None	11	29
	High school	14	37
	bachelor	8	21
	Masters and above	5	13
Age (WHO classification )	Young age	15	39.4
	Middle age	14	36.84
	Elderly	9	23.68
Smoking status	No	37	97.4
	Yes	1	2.6

**Table 2:** Frequencies of asthma control, GERD control, OSA risk, and rhinitis/rhinosnusitis. GERD, gastro-esophageal reflux disease; OSA, obstructive sleep apnea

Asthma control	Frequency	Percent %
Controlled (20-25)	13	34.2
Partially controlled (16-19)	9	23.7
Uncontrolled (16)	16	42.1
Total	38	100
GERD Control		
Controlled or no GERD	20	53
Uncontrolled GERD	18	47
Total	38	100
OSA risk (STOPBANG score)		
Low risk (0-2)	22	57.9
Intermediate risk (3-4)	9	23.7
High risk (5-8)	7	18.4
Total	38	100
Rhinitis/Rhinosinusitis		
Controlled or no Rhinitis/Rhinosunusitis	7	18.4
Uncontrolled Rhinitis/Rhinosinusitis	31	81.6
Total	38	100

**Table 3:** Asthma control (two groups) and STOP-BANG score of OSA risk (two groups) crosstabulation. OSA, obstructive sleep apnea

Asthma control (two group)	N	Risk of OSA low risk (0-2) (n=22)	Risk of OSA intermediate (3-4) to high risk (5-8) (n=16)	p-value
Controlled (20-25)	13	10 (77%)	3 (23%)	0.09
Partially controlled (16-19)/Uncontrolled (<16)	25	12 (48%)	13 (52%)	0.09

**Table 4:** Asthma control (two groups) and CRS control crosstabulation. CRS, chronic rhinosinusitis

Asthma control (two groups)	N	CRS (n=31)	Without CRS (n=7)	p- value
Controlled (20-25)	13	10 (32%)	3 (43%)	0.59
Partially controlled (16-19)/Uncontrolled (<16)	25	21 (68%)	4 (57%)	0.59

**Table 5:** Asthma control (two groups) and GERD control crosstabulation. GERD, gastroesophageal reflux disease

Asthma control (two groups)	N	Controlled GERD (n=20)	Uncontrolled GERD (n=18)	p- value
Controlled (20-25)	13	9 (69%)	4 (31%)	0.14
Partially controlled (16-19)/Uncontrolled (<16)	25	11 (44%)	14 (56%)	0.14

## Discussion

To the best of our knowledge, this is the first study assessing retrospectively severe asthma patients on biologics for appropriateness of biologic treatment prescription. This was mainly by looking at the presence of controllable factors associated with poor asthma control. As per asthma guidelines, diagnosing severe asthma needs careful attention to rule out alternative diagnoses and managing morbidities associated with poor asthma control. Also, assessment of patients while applying a step-wise management approach should be regularly done. Our research hypothesized that those measures were not properly followed, so, unnecessary upgrading of asthma treatment was done, or comorbidities remained unaddressed.

A prospective study included 547 participants concluded that asthma patients had a 39% increased risk of OSA after exclusion of other risk factors such as smoking and obesity. Furthermore, asthma symptoms can be improved by controlling the OSA [16-17]. More than half of our severe asthma sample had moderate or high OSA risk which might have led to poor asthma control and upgrading of treatment.

The GERD on the other hand is very closely linked to asthma which goes side by side with our results [18]. Half of our asthmatic patients had uncontrolled GERD which again could have led to unnecessary upgrading of asthma management. Interestingly more than 80% of our patients had an uncontrolled allergic rhinitis/rhinosinusitis. This may have led to the increased number of uncontrolled asthma patients despite the use of biologic therapy, together with aforementioned factors. It was noticed that most of our patients thought they are capable of using their inhalers properly, however, only half of them were found able to use asthma inhalers properly enough (when asked by investigators to demonstrate it). This might have been secondary to either doctor’s factors (like lack of knowledge or lack of time to teach the patients) or patient’s factors (like low socioeconomic or educational level). Based on above findings, we hypothesize that if all above factors were taken in to consideration when treating severe asthma patients, they would achieve better asthma control and probably some of them might not need biologic therapy. Limitation to our study: The small sample size, and the research was confined to a single center in Riyadh city, and being conducted retrospectively. We would suggest a prospective kind of studies to include larger number in a multicenter approach of severe asthma patients who follow them while controlling all of the factors associated with poor asthma control, to see how many of them would achieve better control, and how many would be controlled with less asthma medications.

## Conclusions

We reassessed our severe asthma patients (those on biologic treatment) for appropriateness of biologic therapy prescribing, mainly by looking at the presence of controllable factors known to exacerbate asthma. We found that significant number of them had at least one comorbid condition that might be interfering with their asthma condition. Also half of them were found not using their asthma medication properly enough. By taking appropriate measures to management and controlling those comorbid conditions and also educating patients about technique to use inhalers might show notable improvement and control in asthmatic patient’s condition. Thereby, reducing the need of upgrading to biologic therapy.

## References

[1] McGregor MC, Krings JG, Nair P, Castro M (2019). Role of biologics in asthma. Am J Respir Crit Care Med.

[2] To T, Stanojevic S, Moores G, Gershon AS, Bateman ED, Cruz AA, Boulet LP (2012). Global asthma prevalence in adults: findings from the cross-sectional world health survey. BMC Public Health.

[3] Boonpiyathad T, Sözener ZC, Satitsuksanoa P, Akdis CA (2019). Immunologic mechanisms in asthma. Semin Immunol.

[4] Schatz M, Rosenwasser L (2014). The allergic asthma phenotype. J Allergy Clin Immunol Pract.

[5] Chung KF, Wenzel SE, Brozek JL (2014). International ERS/ATS guidelines on definition, evaluation and treatment of severe asthma [Internet]. Eur Respir J.

[6] (2019). British Thoracic Society and Scottish Intercollegiate Guidelines Network. British guideline on the management of asthma. A national clinical guideline [Internet]. https://www.brit-thoracic.org.uk/quality-improvement/guidelines/asthma/.

[7] Slejko JF, Ghushchyan VH, Sucher B, Globe DR, Lin SL, Globe G, Sullivan PW (2014). Asthma control in the United States, 2008-2010: indicators of poor asthma control. J Allergy Clin Immunol.

[8] (2021). Global Initiative for Asthma. 2021 GINA report, global strategy for asthma management and prevention (2021 update). https://ginasthma.org/gina-reports.

[9] Braido F, Chrystyn H, Baiardini I (2016). "Trying, But Failing" - the role of inhaler technique and mode of delivery in respiratory medication adherence. J Allergy Clin Immunol Pract.

[10] (2018). Global Initiative for Chronic Obstructive Pulmonary Disease (GOLD). Global strategy for the diagnosis, management, and prevention of chronic obstructive pulmonary disease [Internet]. https://goldcopd.org/archived-reports/.

[11] Rootmensen GN, van Keimpema AR, Jansen HM, de Haan RJ (2010). Predictors of incorrect inhalation technique in patients with asthma or COPD: a study using a validated videotaped scoring method. J Aerosol Med Pulm Drug Deliv.

[12] Kim SY, Sim S, Choi HG (2017). Active, passive, and electronic cigarette smoking is associated with asthma in adolescents. Sci Rep.

[13] Bracken M, Fleming L, Hall P (2009). The importance of nurse-led home visits in the assessment of children with problematic asthma. Arch Dis Child.

[14] Kong DL, Qin Z, Shen H, Jin HY, Wang W, Wang ZF (2017). Association of obstructive sleep apnea with asthma: a meta-analysis. Sci Rep.

[15] Beuther DA, Sutherland ER (2007). Overweight, obesity, and incident asthma: a meta-analysis of prospective epidemiologic studies. Am J Respir Crit Care Med.

[16] Teodorescu M, Barnet JH, Hagen EW, Palta M, Young TB, Peppard PE (2015). Association between asthma and risk of developing obstructive sleep apnea. JAMA.

[17] Alkhalil M, Schulman E, Getsy J (2009). Obstructive sleep apnea syndrome and asthma: what are the links?. J Clin Sleep Med.

[18] Mastronarde JG (2012). Is there a relationship between GERD and asthma?. Gastroenterol Hepatol.

